# The pathophysiology of Peyronie's disease: beyond the Smith's space

**DOI:** 10.1590/S1677-5538.IBJU.2015.06.02

**Published:** 2015

**Affiliations:** Departamento de Urologia, Universidade Federal de Ciências da Saúde de Porto Alegre, Brasil, Av Soledade, 569 / 907B, Porto Alegre, RS, Brasil, Telephone: + 55 51 3378-9995, E-mail: drgraziottin@hotmail.com

Peyronie's disease (PD) is a benign condition clinically characterized by penile nodules and fibrosis of the tunica albuginea (1). The result is penile bending and erectile dysfunction in some patients. Until now urologists can only offer treatments in the late phase of the disease, after the installation of fibrosis. On the other hand, an intriguing course of inflammation precludes the fibrosis of the tunica. In PD this phase is not well studied. Understanding the initial phase, the induction of the inflammation, and the pathway until fibrosis could lead to the development of a treatment that prevents the fibrotic phase in PD.

What is the etiology of PD? It is accepted that penile trauma is an inductor of PD, and the splitting of internal and external tunica layers could accumulate extravascular blood, creating a milieu to inflammation and aberrant wound healing (delamination hypothesis) (2). Moreover, the entrapment of inflammation could perpetuate the process and increase fibrosis (3). TGF beta 1 and myofibroblasts are important steps in PD pathophysiology (4). However, this hypothetical model has not been thoroughly explained.

It is very difficult to study the pathological anatomy of the early steps of PD due to ethical issues, thus we have to retrieve information from former studies. In 1966, Smith (5) revised the histology of 26 cases of PD and compared with 30 cases of autopsies. In the normal anatomy, a space of “vascular, loose, areolar connective tissue sleeve” without elastic fibers separates the corpus cavernosum from the tunica albuginea (Smith's space). In patients with PD, data regarding the onset of the disease was obtained in 21 patients, while 13 patients had less than 6 months of symptoms. The histology varied in function of time of symptom onset; less than 3 months of symptoms presented with inflammatory cellular infiltrate, while longer lesions more fibrosis and ossification. The inflammatory process with lymphocytes and plasma cells in early lesions was located in the areolar connective tissue of Smith's space. The inflammation was perivascular, occasionally with endothelial proliferation and perivascular fibrosis. More advanced cases demonstrated fibrosis of the connective tissue in the Smith's space, advancing on the cavernous tissue and destroying the smooth muscle bundles in the intercavernous septum. Ossification was also located in the Smith's space.

Recently, new discoveries regarding the initiation of the inflammatory process in other tissues paved the way to understand the pathophysiology of PD in the early phase. Based upon this body of evidence we can build a theoretical model to explain in part the pathophysiology of PD.

The first step is the vascular inflammation in the Smith's space or intersinusoidal space. After trauma or other stimuli, endothelial pro-inflammatory modification occurs. Post-capillary venous endothelial cells are more prone to this reaction. The “sterile” inflammatory response is initiated mostly by exposure of innate immune cells, primarily macrophages, dendritic cells and neutrophils, or damage-associated molecular patterns (DAMPs) (6). This results in the secretion of inflammatory cytokines IL-1β, IL-6 and TNF-α, and many different chemokines. Several molecules can act as DAMPs including hyaluronan, heparin sulfate, heat shock proteins, and ATP. The vascular endothelium expresses chemoattractants and adhesion molecules in response to these proinflammatory stimuli. Neutrophils in circulation are recruited due to action of E-selectin, P-selectin and integrins (VCAM-1, ICAM-1 and 2) and migrate to the site of tissue damage under the influence of CD99, PECAM-1, VE-Cadherin and ICAM-1 (6). Another important step to complement this process of inflammation is platelet aggregation and local coagulation (7). Trauma can damage the endothelium and expose subendothelial molecules that promote platelet aggregation via P-selectin. Immunothrombosis in veins of the subtunical space could amplify the process of inflammation. Interestingly, uncontrolled coagulation is implied in inflammation and fibrosis in liver, heart, kidney, and lungs (8). Those processes could recur several times, contributing for phases of recrudescence and resolution of inflammation. Also, it is possible that in some areas inflammation and fibrosis are in different phases, leading to the conclusion that PD is in continuing remodeling.

As discussed before, the Smith's space and the surrounding corpus cavernosum are ideal sites for the development of PD under this model. The fibrosis advancing the intersinusoidal space could also be explained. It is very unlikely that PD occurs only inside the tunica albuginea and septum as in the delamination model.

The importance of the proposed model is that we can envision several experiments to test the endothelium, the immune system and the hemostasis as contributors to the inflammation in the development of PD. Genetic variations and differences in the expression of molecules under the optics of this model could identify a cohort of predisposed population for PD. If we could control the inflammation or even avoid it, a prophylaxis of PD would be possible.

## References

[1] Gholami SS, Gonzalez-Cadavid NF, Lin CS, Rajfer J, Lue TF (2003). Peyronie's disease: a review. J Urol.

[2] Devine CJ, Somers KD, Jordan SG, Schlossberg SM (1997). Proposal: trauma as the cause of the Peyronie's lesion. J Urol.

[3] Lue TF (2002). Peyronie's disease: an anatomically-based hypothesis and beyond. Int J Impot Res.

[4] Gonzalez-Cadavid NF (2009). Mechanisms of penile fibrosis. J Sex Med.

[5] Smith BH (1966). Peyronie's disease. Am J Clin Pathol.

[6] Williams MR, Azcutia V, Newton G, Alcaide P, Luscinskas FW (2011). Emerging mechanisms of neutrophil recruitment across endothelium. Trends Immunol.

[7] Engelmann B, Massberg S (2013). Thrombosis as an intravascular effector of innate immunity. Nat Rev Immunol.

[8] Mercer PF, Chambers RC (2013). Coagulation and coagulation signalling in fibrosis. Biochim Biophys Acta.

